# Critical ACE2 Determinants of SARS-CoV-2 and Group 2B Coronavirus Infection and Replication

**DOI:** 10.1128/mBio.03149-20

**Published:** 2021-03-16

**Authors:** Lily E. Adams, Kenneth H. Dinnon, Yixuan J. Hou, Timothy P. Sheahan, Mark T. Heise, Ralph S. Baric

**Affiliations:** a Department of Microbiology & Immunology, University of North Carolina at Chapel Hill, Chapel Hill, North Carolina, USA; b Department of Epidemiology, University of North Carolina at Chapel Hill, Chapel Hill, North Carolina, USA; c Department of Genetics, University of North Carolina at Chapel Hill, Chapel Hill, North Carolina, USA; d Rapidly Emerging Antiviral Drug Discovery Initiative, University of North Carolina, Chapel Hill, North Carolina, USA; Johns Hopkins Bloomberg School of Public Health

**Keywords:** COVID-19, SARS-CoV-2, coronavirus, host range, receptors, virus-host interactions

## Abstract

The angiotensin-converting enzyme 2 (ACE2) receptor is a major severe acute respiratory syndrome coronavirus 2 (SARS-CoV-2) host range determinant, and understanding SARS-CoV-2-ACE2 interactions will provide important insights into COVID-19 pathogenesis and animal model development. SARS-CoV-2 cannot infect mice due to incompatibility between its receptor binding domain and the murine ACE2 receptor. Through molecular modeling and empirical *in vitro* validation, we identified 5 key amino acid differences between murine and human ACE2 that mediate SARS-CoV-2 infection, generating a chimeric humanized murine ACE2. Additionally, we examined the ability of the humanized murine ACE2 receptor to permit infection by an additional preemergent group 2B coronavirus, WIV-1, providing evidence for the potential pan-virus capabilities of this chimeric receptor. Finally, we predicted the ability of these determinants to inform host range identification of preemergent coronaviruses by evaluating hot spot contacts between SARS-CoV-2 and additional potential host receptors. Our results identify residue determinants that mediate coronavirus receptor usage and host range for application in SARS-CoV-2 and emerging coronavirus animal model development.

## Abstract (precis)

SARS-CoV-2 (the causative agent of COVID-19) is a major public health threat and one of two related coronaviruses that have caused epidemics in modern history. A method of screening potential infectible hosts for preemergent and future emergent coronaviruses would aid in mounting rapid response and intervention strategies during future emergence events.

## OBSERVATION

Since its emergence in December 2019, severe acute respiratory syndrome coronavirus 2 (SARS-CoV-2) has infected tens of millions, leading to over 2 million deaths worldwide (1, 2). SARS-CoV-2, SARS-CoV (3), and other related group 2B coronaviruses poised for emergence (4) primarily utilize cellular angiotensin-converting enzyme 2 (ACE2) via the viral receptor binding domain (RBD) for entry. Zoonotic coronaviruses like SARS-CoV-2 emerge following a transmission event between reservoir species and a new permissive host where factors like RBD-receptor interactions and host proteases support infection. Although emergent and preemergent coronaviruses have been demonstrated to utilize ACE2 from multiple species in addition to the purported natural host, the mechanisms of these interactions are largely unknown, complicating reservoir species identification and disease potential in mammals. The identification and characterization of key molecular interactions between specific RBD and ACE2 molecules could be applied to predict and determine the plasticity of the virus host range.

In addition to providing new insights into coronavirus receptor interactions and host range, characterizing determinants of ACE2 species restriction also has implications for SARS-CoV-2 animal model development. Mouse models have been essential for understanding the pathogenesis of coronaviruses and have been key resources for the preclinical development of vaccines and antiviral therapies (5–7). However, standard laboratory mice are not permissive to SARS-CoV-2 infection due to incompatible interactions between the RBD and the mouse ACE2 receptor (mACE2) (8), which is a substantial barrier to vaccine and antiviral development. While human ACE2 (hACE2) mouse models (9–13) and mouse-adapted viruses (14–16) have been developed, limitations exist. For example, hACE2 transgenic mice are permissive for nonadapted SARS-CoV-2 viruses, but the pathogenesis in these mice, with mortality driven by virus-induced encephalitis and multiorgan infection, is not representative of that observed in humans (10, 11, 13). Additionally, mouse-adapted viruses contain amino acid changes and do not fully reflect circulating virus strains. The development of immunocompetent models that more faithfully model human pathogenesis will facilitate more robust and rigorous evaluations of vaccines, antibodies, and therapeutics. To map the SARS-CoV-2 RBD and mACE2 interaction network, we created a panel of mACE2 receptors with increasing levels of humanizing mutation based on predictive structural modeling, thus identifying the minimum changes necessary to restore replication.

**FIG 1:**
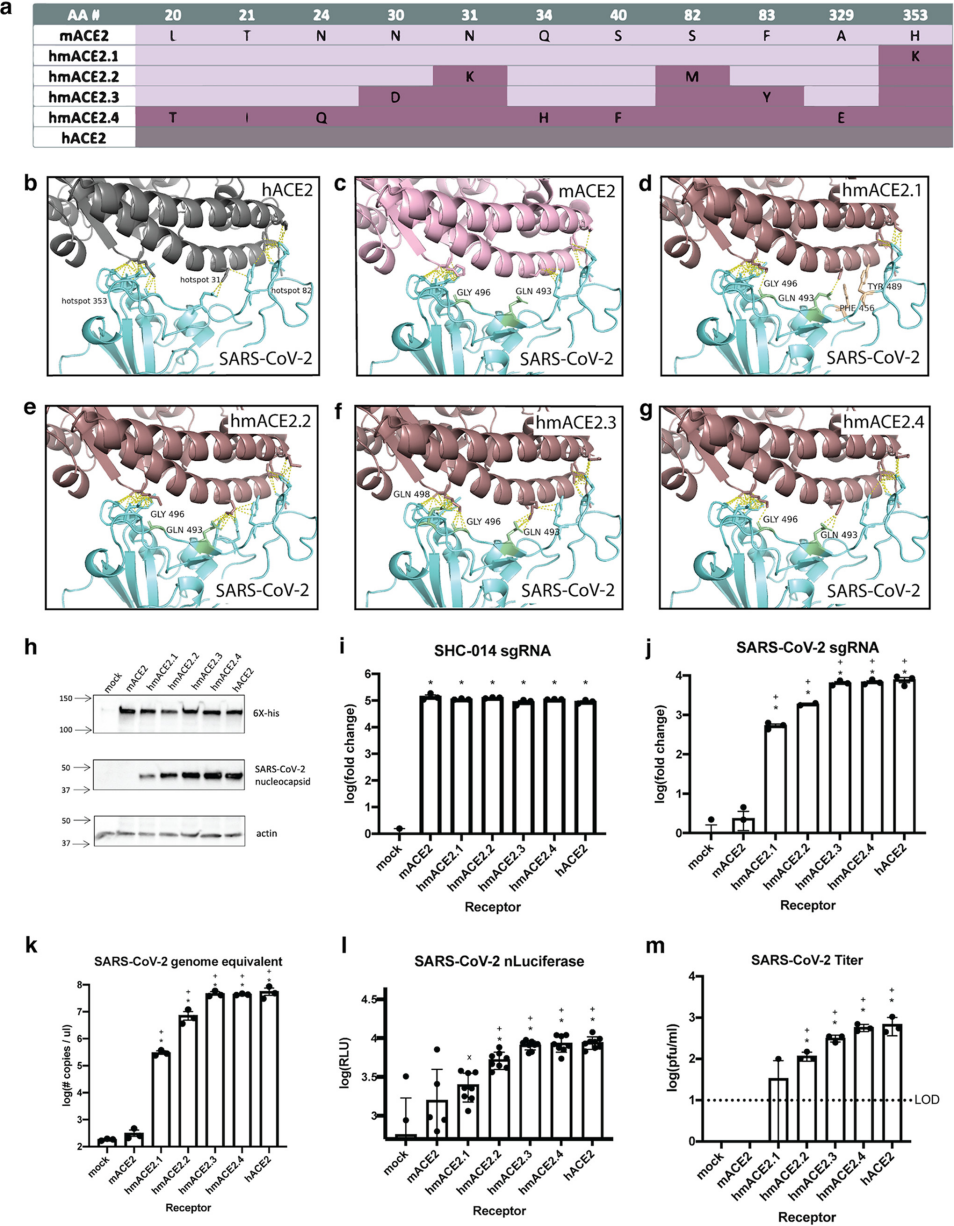
Engineered mutations in the mACE2 background reestablish contacts with SARS-CoV-2 RBD and support SARS-CoV-2 infection and replication. (a) Residues modified to incrementally increase humanization of mACE2 (blue, mouse; pink, human). (b) Contacts between the SARS-CoV-2 RBD and the three hACE2 interaction hot spots. (c) Lost contacts between mACE2 hot spots and SARS-CoV-2 RBD Q493/G496 (red). (d to g) Key contacts gained and lost through mACE2 humanization of one amino acid change (Q493 and G496 gained, F456 and Y489 lost) (d), three amino acid changes (Q493, F456, and Y489 maintained, G496 lost) (e), five amino acid changes (all regained) (f), and a swap of the entire hACE2 binding interface (all maintained) (g). (h) Western blotting confirming ACE2 receptor expression (anti-6×-His) and SARS-CoV-2 (anti-nucleocapsid) infection. (i) qPCR 24 h after infection by SARS-like virus SHC014. (j) qPCR. (k) Genome equivalents. (l) Luciferase. (m) Titer 24 h following virus infection. *n* = 3 replicates per sample. Data analyzed by 2-way analysis of variance (ANOVA) followed by Dunnett’s multiple comparisons. Error bars represent standard error about the mean. *, comparisons to mock where *P* ≤ 0.0001; x, comparisons to mock where *P* ≤ 0.001; +, comparisons to mACE2 where *P* ≤ 0.0001. sgRNA, single guide RNA.

Based on previous SARS-CoV research and published structures (17, 18), we identified likely SARS-CoV-2 RBD and ACE2 interactions via molecular modeling experiments. The ACE2 interaction residues support the presence of three hot spots: position K353 interacts with SARS-CoV-2 binding residues G496, N501, and Y505, position K31 forms a salt bridge with ACE2 residue K353 and interacts with SARS-CoV-2 Q493 and Y489, and position M82 interacts with RBD residues F486, N487, and Y489 (Fig. 1a and b). These interface hot spots are critical molecular constraints for receptor interaction and entry, and divergent residues at these sites are predicted to significantly decrease binding between mACE2 and SARS-CoV-2 RBD (8).

In order to evaluate the impact of mACE2 humanization at each of these interaction hot spots, we first modeled the interaction between SARS-CoV-2 RBD and mACE2 (Fig. 1c). From this, we predict that molecular incompatibility between the SARS-CoV-2 RBD and mACE2 may result in a profound loss of contact between mACE2 and the SARS-CoV-2 RBD residues Q493 and G496. We predict this significantly contributes to receptor incompatibility. We then modeled mutant mACE2 receptors that contain human residues that are predicted to reestablish increasing degrees of contacts, termed hmACE2.1 through hmACE2.4 (Fig. 1d to g) to identify and predict the changes that would mitigate this contact loss. We predict that the single amino acid change H353K in mACE2 can reestablish lost contacts (hmACE2.1). However, this mutation may inappropriately remodel contact between mACE2 hot spot residue 31 and SARS-CoV-2 RBD F456 and Y489 residues, leading to loss of function. Mutations of N31K, S82M, and H353K in the mACE2 backbone (hmACE2.2) are predicted to reestablish the contacts with those lost from H353K but fail to maintain contact with G496. By introducing an additional two mutations to balance charges and conformations, N30D and F83Y (hmACE2.3), all lost contacts are predicted to be reestablished, producing a fully humanized mACE2. Additionally in this model, SARS-CoV2 RBD-ACE2 interactions reestablished by the above five amino acid changes (N30D, N31K, S82M, F83Y, and H353K) in hmACE2.3 are the same contacts reestablished by a swap of the entire mACE2 binding interface with that of hACE2 (hmACE2.4).

To experimentally test these models, a panel of hmACE2 receptors was generated to directly evaluate whether these predicted contact residues are essential for SARS-CoV-2 infection. The receptors were introduced into mouse delayed brain tumor astrocytoma (DBT-9) cells (19, 20), normally nonpermissive for SARS-CoV-2 infection, and the cells were then infected with SARS-CoV-2 to test each chimeric receptor’s ability to mediate infection. Expression of each receptor and detection of viral particles were confirmed by Western blotting (Fig. 1h). Infection of the receptor panel by preepidemic SARS-like bat CoV SHC014, which does not grow in unmodified DBT cells (4, 21) but utilizes both mACE2 and hACE2 equally, was employed as a functional positive control (Fig. 1i). We found that all of the receptors supported SHC014 infection equivalently, indicating functionally equivalent receptor surface expression. The viruses used were derived from molecular clones that, due to the proofreading capability of the coronavirus Nsp14, undergo low rates of mutation (22, 23). Consequently, our study was designed to only sample after a single round of replication and is unlikely to amplify, detect, or be influenced by rare variants that emerge due to binding effect. As quantified by quantitative PCR (qPCR) and viral titer, we found that all humanized receptors supported SARS-CoV-2 entry and replication, while wild-type (WT) mACE2 did not. We found that mACE2 facilitated isolated instances of SARS-CoV-2 entry as quantified by luciferase assay, but the positivity is likely due to background luminescence emitted during the assay. While a single change at position 353 was sufficient to support SARS-CoV-2 infection by the mACE2 molecule, all five amino acid changes balancing the three binding hot spots were required to support efficient infection at levels comparable to hACE2, suggesting that satisfaction of interactions with the three hot spots are required for maximal infection. There was no significant increase in infection of hmACE2 remodeled to reflect the entire hACE2 binding interface past the remodeling of the five hot spot residues (Fig. 1j to m). As such, we determined that additional mutations past the five to satisfy the hot spots were not necessary.

**FIG 2:**
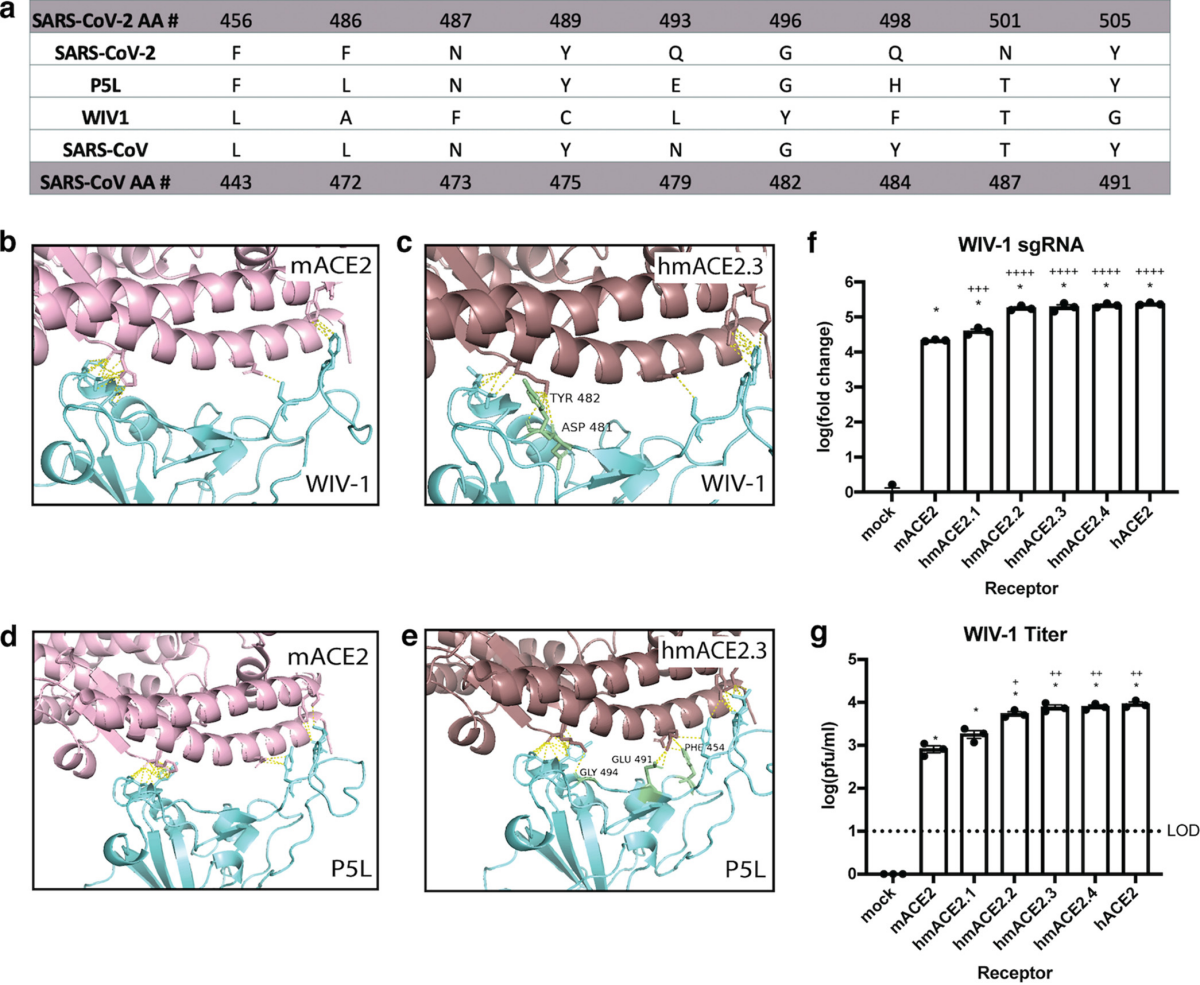
hmACE2 contacts with WIV-1 and P5L are predicted to support replication. (a) Aligned contact residues of SARS-CoV-2, SARS-CoV, WIV-1, and P5L. (b and c) Contacts identified between mACE2 and WIV-1 (b) and those gained between hmACE2 and WIV-1 (c) include Y482 and D481. (d and e) Contacts identified between mACE2 and P5L (d) and gained between hmACE2 and P5L (e) include G496, E493, and F456 (right). (f and g) qPCR (f) and titer (g) 24 h following WIV-1 infection. Data analyzed by 2-way ANOVA followed by Dunnett’s multiple comparisons. Error bars represent standard error about the mean. *, comparisons to mock where *P* ≤ 0.0001. Plus signs represent comparisons to mACE2 (+, *P* ≤ 0.05; ++, *P* ≤ 0.01; +++, *P ≤* 0.001; ++++, *P* ≤ 0.0001).

Given the importance of the three ACE2 interaction hot spots for SARS-CoV-2 infection, we asked whether these same determinants were important for infection by other preepidemic group 2B coronaviruses. We modeled interactions between hmACE2.3 and two related group 2B CoVs, preepidemic SARS-like bat CoV WIV-1 (4) and pangolin CoV, designated P5L (Fig. 2a), a group 2B pangolin virus closely related to SARS-CoV-2 (24). Our analysis indicates that, though the RBDs of the viruses are divergent past the hot spot-interacting residues (Fig. S3), both the P5L and WIV-1 RBD likely interact with the three ACE2 hot spots predicted to mediate receptor binding, indicating that these interactions may be generally important for receptor binding by SARS-CoV- and SARS-CoV-2-like viruses (Fig. 2b to e). To further test this, we evaluated our panel of chimeric mACE2 receptors for their ability to enhance WIV-1 murine receptor usage. Consistent with earlier findings (4), we show that WIV-1 can utilize mACE2; however, the presence of the 5 amino acid changes that restored the three binding hot spots significantly increased WIV-1 infection yields to levels that were comparable to cells expressing human ACE2 (Fig. 2f to g). Though WIV-1 replicated to higher titers in this assay than SARS-CoV-2, there are other differences between these viruses that may result in enhanced replication by WIV-1. Therefore, the logical interpretation is that the presence of the humanized chimeras resulted in higher rates of infection over mACE2. This indicates that these interaction hot spots are broadly important for receptor recognition by SARS-CoV- and SARS-CoV-2-like group 2B coronaviruses and that the interactions predicted are favorable for productive infection.

10.1128/mBio.03149-20.3 FIG S3 Structure-based multiple sequence alignment of the RBD from SARS-CoV, SARS-CoV-2, WIV-1, and P5L. Download FIG S3, TIF file, 1.6 MB. Copyright © 2021 Adams et al. 2021 Adams et al. https://creativecommons.org/licenses/by/4.0/ This content is distributed under the terms of the Creative Commons Attribution 4.0 International license.

Recent work has identified ACE2 variants from myriad mammalian species that support SARS-CoV-2 infection (25, 26), both for model development and as potential reservoirs. Though ACE2 variant sequences are divergent, we asked whether the sequence homology was sufficient to model the interactions between these host receptors and the SARS-CoV-2 RBD. Based on pairwise sequence alignments to hACE2 (Fig. S2), we modeled the RBD interaction with ACE2 molecules from species reported to support entry and infection *in vitro* (ferret, macaque, mink, cat, pig, guinea pig, and dog) as well as those from species reported to support little to no infection (mouse, chicken) (Fig. S1) (25, 27). The molecular interactions, both favorable and unfavorable, we predicted align with the differences in infection identified in recent work. For example, though human and ferret ACE2 differ at residue 81, we predict the SARS-CoV-2 RBD still interacts favorably with ferret ACE2 to satisfy the three critical interaction hot spots. Conversely, though chicken ACE2 contains hot spot residue K353, we predict molecular incompatibility due to lack of contact at hot spot 31. Furthermore, ACE2 molecules from other highly susceptible species are homologous in some (cats) or all (nonhuman primates) hot spot residues, which enable contact between the three hot spots and the SARS-COV-2 RBD (8), providing further support that the hot spot analysis is highly informative. Our predictions indicate that the three hot spots may be used to accurately predict the receptor interaction between ACE2 and group 2B coronaviruses and that the sequence homology between ACE2 molecules is sufficient to model these interactions in instances where no experimentally determined structure is available. This enables a molecular modeling-based screening approach to host range predictions. Furthermore, while additional work has found that some animals predicted to be reservoirs to SARS-CoV-2 infection are not permissive *in vivo* (27, 28), all of the animals predicted to not be infectible based on receptor interaction have been identified as nonpermissive hosts. This shows that, while favorable receptor interaction may not be sufficient for productive infection, it is a primary determinant of host range. Additionally, this work demonstrates that this contact analysis can be used to rapidly filter ACE2 molecules from different species to identify or eliminate potential reservoir hosts or animal models.

10.1128/mBio.03149-20.1 FIG S1 ACE2 hotspot contacts are predictive of SARS-CoV-2 host range. (a) Aligned hotspot residues of the ACE2 molecule from humans, macaques (Macaca mulatta), ferrets (Mustela putorius), mink (Mustela lutreola), domestic cats (Felis catus), pigs (Sus scrofa), guinea pigs (Cavia porcellus), dogs (Canis lupus), mice (Mus musculus), and chickens (Gallus gallus). (b to h) Predicted hotspot contacts between SARS-CoV-2 RBD and the ACE2 molecule from humans (b), macaques (c), ferrets (d), mink (e), cats (f), pig (g), guinea pig (h), dog (i), mouse (j), and chicken (k). (l and m) Predicted hotspot contacts between SARS-CoV-2 variant Y453F and mink (l) and human (m) ACE2. (n) Predicted hotspot contacts between SARS-CoV-2 variant N501Y and human ACE2. Download FIG S1, TIF file, 1.2 MB. Copyright © 2021 Adams et al. 2021 Adams et al. https://creativecommons.org/licenses/by/4.0/ This content is distributed under the terms of the Creative Commons Attribution 4.0 International license.

10.1128/mBio.03149-20.2 FIG S2 Selected pairwise sequence alignments for human ACE2 against the ACE2 from ferret (a), pig (b), guinea pig (c), dog (d), chicken (e), and mouse (f). Download FIG S2, TIF file, 0.7 MB. Copyright © 2021 Adams et al. 2021 Adams et al. https://creativecommons.org/licenses/by/4.0/ This content is distributed under the terms of the Creative Commons Attribution 4.0 International license.

Our results provide key insights into the amino acid differences between human and mouse ACE2 molecules that are responsible for determining SARS-CoV-2 receptor activity and species specificity. In mouse-adapted viruses, mACE2 binding was restored by the single mutations Q498H (29), N501Y (16), Q498Y (14), and Q493K (15), demonstrating several pathways to increased mACE2 usage. Recent work has also identified various isolates able to infect mink that contain RBD substitution Y453F, which likely improves contact with hot spot 31 in the mink ACE2 molecule and appear to retain contact with all hot spots in human ACE2 (30). Furthermore, emerging variants from Africa and London contain substitution N501Y, an RBD substitution that likely improves contact with both mouse ACE2 and human ACE2, due to biochemical and structural property changes at hot spot 353. These data (supplemental material) support our findings that interactions at single hot spots enhance usage, leading to multiple evolutionary pathways to adaptation, but efficient adaptation may require multiple mutations that allow for efficient usage of multiple hot spots. We also identified key contact residues within ACE2 that mediate receptor interactions with other epidemic and preepidemic group 2B coronaviruses. Given the importance of these interactions for SARS-CoV-2 infection, we suggest that these determinants may be used to also predict susceptible species for model development or reservoir formation. Furthermore, it should be possible to introduce these changes into the mACE2 gene *in vivo* by gene editing (31), thereby generating a chimeric ACE2 receptor with physiologic expression patterns that is capable of mediating infection by clinical isolates of SARS-CoV-2 and potentially preemergent group 2B coronaviruses. This work did not address, however, the influence of ACE2 isoforms or allelic variants on host susceptibility, including cytokine-inducible ACE2 variants or variation in ACE2 expression levels (32, 33). In addition to isoform expression, host factors like protease state also significantly impact cellular susceptibility to SARS-CoV-2 (34, 35), as evidenced by conflicting results between *in vitro* assays and *in vivo* infection to identify potential SARS-CoV-2 reservoir species and animal models (25, 26, 28). We have found that favorable receptor interaction is absolutely necessary for productive infection, consonant with earlier findings (36), but all potential hosts must be fully evaluated in downstream analysis to investigate additional factors determining host range.

## 

### Molecular modeling and visualization.

The ACE2 panel was aligned using Geneious Prime (version 2020.0.5). The hACE2 sequence is found under GenBank accession no. BAB40370 and mACE2 sequence under GenPept accession no. NP_081562. Scores were computed using BLOSUM62 scoring matrix. Three-dimensional structures were predicted using MODELLER homology modeling tool (37) from pairwise sequence alignment to the reference SARS-CoV-2 RBD hACE2 complex (PDB ID 6M0J) and SARS-CoV RBD hACE2 complex (PDB ID 2AJF). WIV-1 RBD was modeled using the reference sequence, GenPept accession no. AGZ48828.1, and P5L RBD was modeled using GenBank accession no. MT040335.1. Additional receptor interactions were modeled from the following translated reference sequences: chicken, GenBank accession no. XM_416822.5; guinea pig, GenBank accession no. XM_023562040.1; ferret, GenBank accession no. XM_004758886.2; pig, GenBank accession no. EU518378.1; dog, GenBank accession no. NM_001165260.1; macaque, GenBank accession no. NM_001135696.1; and mink, GenBank accession no. MT560518.1. Contacts were identified and visualized in the PyMOL molecular graphics system (Schrödinger LLC). Structure-based multiple sequence alignment was generated with UCSF Chimera.

### Viruses and cells.

All viruses were derived using infectious clone technology as previously described, including icSARS-CoV-2-WT (GenBank accession no. MT461669), icSARS-CoV-2-nLuc (GenBank accession no. MT461671) (38), and icWIV1-CoV (4) (GenBank accession no. KF367457.1). Mouse delayed brain tumor astrocytoma (DBT-9) (19, 20) cells were maintained in minimum essential medium (MEM; Gibco), 10% fetal clone II serum (FCII; HyClone), and 1× antibiotic/antimycotic (Gibco). Vero E6 cells were maintained in Dulbecco’s modified Eagle’s medium (DMEM; Gibco), 5% FC11, and 1× antibiotic/antimycotic.

### ACE2 panel construction.

Each mouse, human, and chimeric ACE2 was expressed in pCDNA3.1 vector that conjugates a C-terminal 6×-His tag to the end of the expressed protein. The hmACE2 panel was generated with a combination of site-directed mutagenesis and custom double-stranded gBlocks gene fragments (IDT). DBT-9 cells were transfected using Lipofectamine 2000 transfection reagent (Invitrogen). Expression of each receptor was verified via staining against a 6×-His epitope (Invitrogen; catalog no. PA1-983B) and Western blotting.

### Infection and analysis.

Cells were infected in triplicate 24 h posttransfection for receptor usage analysis at a multiplicity of infection (MOI) of 1 for 1 h. Inoculum was removed, cells were washed with Dulbecco's phosphate-buffered saline (DPBS), and medium was replaced. Receptor usage was analyzed 24 h postinfection. RNA was collected via TRIzol (Invitrogen) and extracted using Direct-zol RNA MiniPrep kit (Zymo Research). Viral RNA was quantified in duplicate via reverse transcriptase quantitative PCR (qRT-PCR) using TaqMan Fast Virus 1-Step Master Mix (Thermo Fisher Scientific) on a QuantStudio 3 (Applied Biosystems). Viral RNA was quantified with a subgenomic diagnostic assay primer set (Table 1). Host 18S rRNA was used as housekeeping control (Invitrogen; catalog no. 4319413E). Viral RNA was analyzed using the threshold cycle (ΔΔ*CT*) method and fold change over viral RNA in empty vector-transfected cells. SARS-CoV-2 genome equivalents were calculated against a standard curve with a diagnostic genomic assay primer set against Nsp4 (Table 1). Receptor expression was confirmed via Western blotting against the 6×-His tag and virus nucleocapsid. For virus titer calculation, Vero E6 cells were infected at an MOI of 1 for 1 h. Inoculum was removed, and the monolayer was washed with PBS and replaced with medium. The medium was removed 24 h postinfection and stored at −80°C until the titer was determined by plaque assay. Briefly, virus was serial diluted and inoculated onto confluent monolayers of Vero E6 cells, followed by agarose overlay. Plaques were visualized on day 2 postinfection via staining with neutral red dye.

**TABLE 1:** Primers and sequences used in this study

Primer	Sequence
SARS-CoV-2 sgN F	5′-TTCGATCTCTTGTAGATCTGTTCTC-3′
SARS-CoV-2 sgN R	5′-GCGTTCTCCATTCTGGTTACT-3′
SARS-CoV-2 sgN probe	5′-FAM-ACGTTTGGTGGACCCTCAGATTCA-3′
SARS-CoV-2 Nsp4 F	5′-GTGCTCATGGATGGCTCTATTA-3′
SARS-CoV-2 Nsp4 R	5′-CGTGCCTACAGTACTCAGAATC-3′
SARS-CoV-2 Nsp4 probe	5′-FAM-ACCTACCTTGAAGGTTCTGTTAGAGTGGT-3′
WIV1-CoV sgN F	5′-CGATCTCTTGTAGATCTGTTCTCTAA-3′
WIV1-CoV sgN R	5′-CTGTGGGTCCACCAAATGTA-3′
WIV1-CoV sgN probe	5′-FAM-CCAATCAAACCAGCGTAGTGCCC-3′

### Data availability.

All relevant data sets generated or analyzed in this study have been included in the article. Additional information, including replicates, is available upon request.
