# Supplementary figures and images for: Critical ACE2 Determinants of SARS-CoV-2 and Group 2B Coronavirus Infection and Replication
Source: mBio. 2021 Mar 16;12(2):e03149-20. doi: 10.1128/mBio.03149-20 (PMC8092278; doi:10.1128/mBio.03149-20)

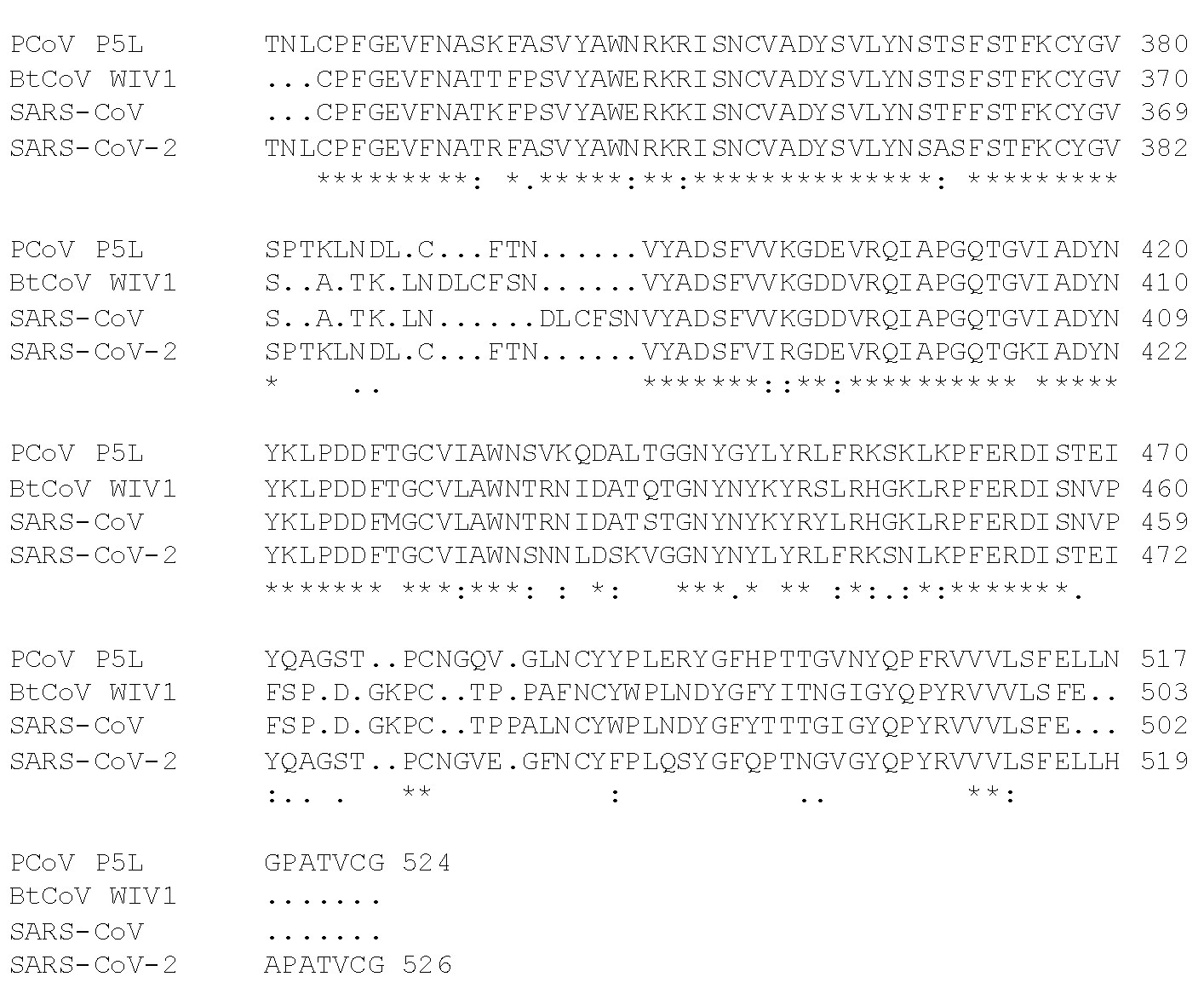

Supplement: FIG S3 [file mBio.03149-20-sf003.tif]

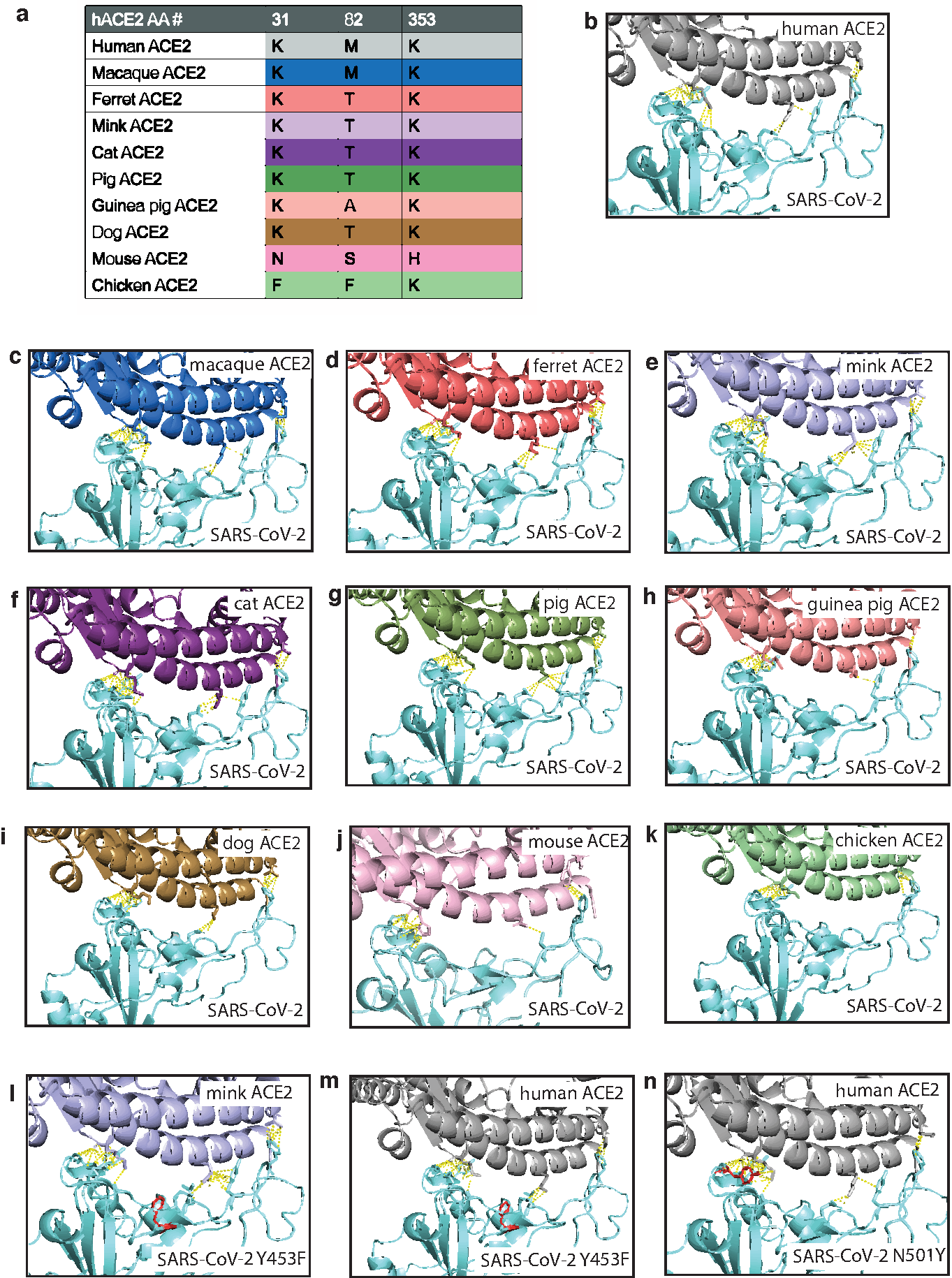

Supplement: FIG S1 [file mBio.03149-20-sf001.tif]

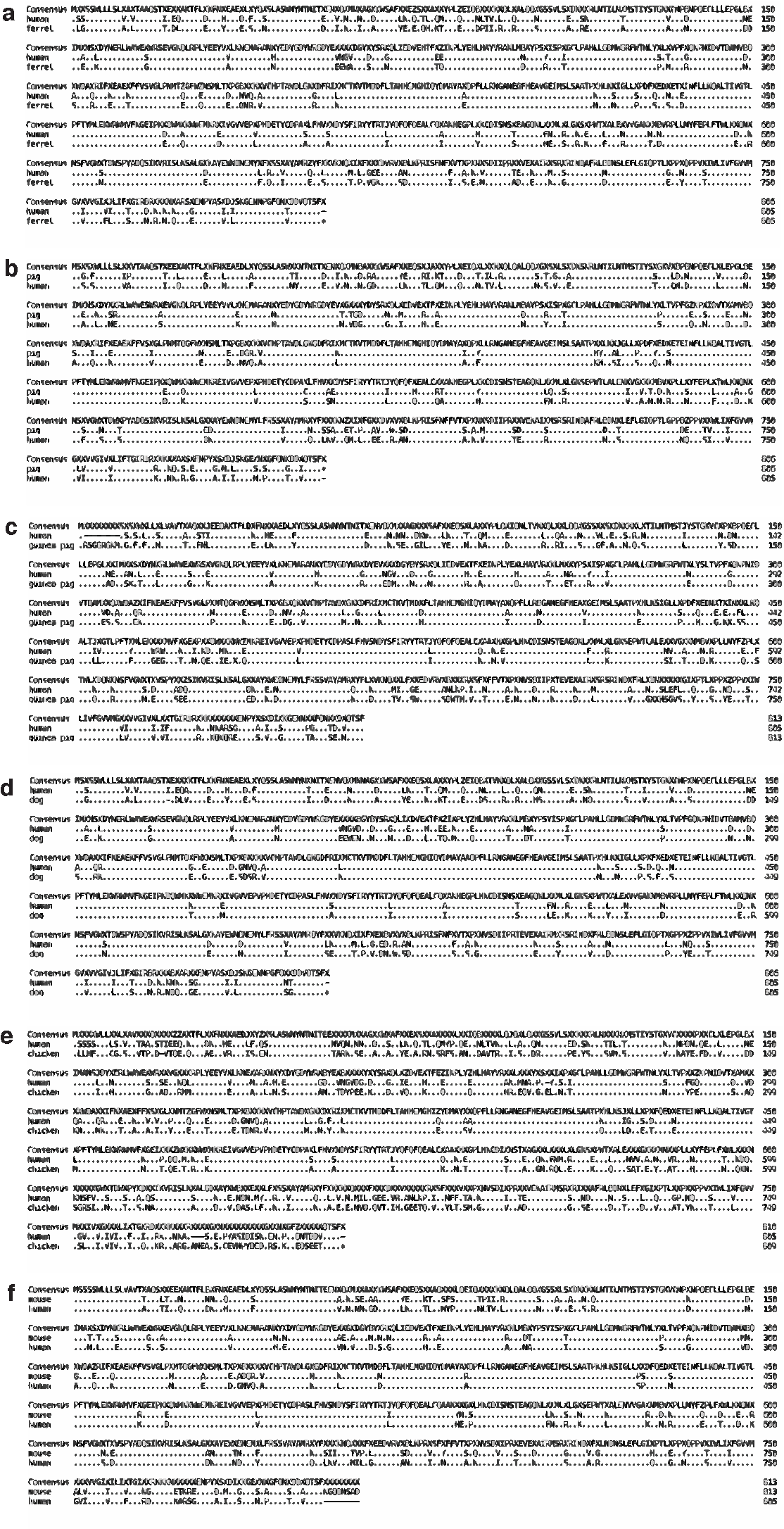

Supplement: FIG S2 [file mBio.03149-20-sf002.tif]
